# Progressive Improvement in Static Glabellar Lines After Repeated Treatment With DaxibotulinumtoxinA for Injection

**DOI:** 10.1097/DSS.0000000000003211

**Published:** 2021-08-16

**Authors:** Richard Glogau, Theda C. Kontis, Yan Liu, Conor J. Gallagher

**Affiliations:** * Department of Dermatology, University of California at San Francisco, San Francisco, California;; † Division of Facial Plastic and Reconstructive Surgery, Department of Otolaryngology-Head and Neck Surgery, Johns Hopkins Medical Institutions, Baltimore, Maryland;; ‡ Revance Therapeutics, Inc, Newark, California

## Abstract

DaxibotulinumtoxinA for Injection (DAXI) is a novel botulinum toxin in development that has demonstrated efficacy on dynamic glabellar lines.

**OBJECTIVE:**

To evaluate the effect of repeated DAXI treatment on static glabellar lines.

**METHODS:**

This post hoc analysis included adults with moderate or severe dynamic glabellar lines who were treated with 40U DAXI in the SAKURA clinical program. Glabellar line severity was evaluated through validated subject and investigator scales.

**RESULTS:**

Overall, 568 subjects received 3 DAXI treatments. Most subjects were White (92.3%) and female (85.7%). At baseline, 9.0% and 27.3% of subjects had no static glabellar lines based on subject and investigator assessment, respectively. Four weeks after DAXI Treatment Cycle 1, the proportion of subjects with no static glabellar lines increased to 57.9% and 64.8% based on subject and investigator assessment, respectively. At Week 4 after DAXI Treatment Cycles 2 and 3, the proportion further increased to 68.7% and 71.5%, respectively, based on subject assessment and 75.0% and 77.6% based on investigator assessment.

**CONCLUSION:**

Subjects who received repeated DAXI treatment showed progressive improvement in their static glabellar lines. The extended duration of therapeutic benefit with DAXI on dynamic glabellar lines likely provides a long period of muscle inactivity/hypoactivity, during which dermal remodeling can occur.

## Abstract 2

Supplemental Digital Content is Available in the Text.

The persistence of dynamic glabellar lines over a prolonged period results in the development of etched-in, static lines due to adaptive structural changes in the dermis caused by the repetitive contraction of the underlying glabellar complex muscles.^1–3^ Botulinum toxin Type A (BoNTA) treatments are commonly used to treat dynamic glabellar lines. Botulinum toxin Type A causes temporary focal chemodenervation of the injected muscles, and a demonstrated secondary benefit of BoNTA treatment on static glabellar lines has been observed, both anecdotally and in clinical trials.^4–6^ It is believed that relaxation of the muscles of the glabellar complex with BoNTA relieves repetitive contraction, allowing an opportunity for the dermis to remodel.^3,4^ In addition, it is believed that the longer the glabellar muscles are weakened by BoNTA treatment, the longer the dermis has to undergo remodeling to gradually soften static glabellar lines. Currently approved BoNTA treatments have a reported duration of effect of 3 to 4 months (12–16 weeks).^7,8^ Despite this, patients only receive 2 BoNTA treatments per year on average,^9^ suggesting that there is a marked period when the muscles of the glabellar complex are active, which would offset any benefits of dermal remodeling that may occur during the denervated period. A BoNTA product with an extended duration of therapeutic benefit on dynamic glabellar lines may show enhanced benefit in the softening of static glabellar lines.

DaxibotulinumtoxinA for Injection (DAXI; Revance Therapeutics, Inc, Newark, CA) is a novel formulation of BoNTA in development for the treatment of moderate or severe dynamic glabellar lines. Unlike BoNTA products currently approved in the United States for the treatment of glabellar lines,^7,8,10,11^ DAXI is created with a unique formulation, which includes a proprietary stabilizing excipient peptide (RTP004) and does not contain human serum albumin. The SAKURA clinical program was the largest Phase 3 clinical program in aesthetics and included 2 pivotal trials, which evaluated the efficacy and safety of a single DAXI treatment,^12,13^ and an open-label study, which evaluated the safety and efficacy of up to 3 DAXI treatments.^14,15^ The SAKURA trials demonstrated that, in patients treated with DAXI, the median time to return to moderate or severe dynamic glabellar line severity was 24 weeks and patients achieved a high degree of clinical efficacy (with peak effect observed between Weeks 2 and 4).^12–14^ In addition, a progressive improvement in patients' static glabellar lines was visually noted by study investigators, including the authors T.C. Kontis and R. Glogau. The objective of this post hoc analysis was to evaluate the effect of repeated BoNTA treatment on static glabellar lines among a subset of subjects who received 3 DAXI treatments in the SAKURA clinical program to understand the potential real-world performance.

## Methods

### Study Design

Two multicenter, randomized, double-blind, placebo-controlled, single-treatment Phase 3 trials (SAKURA 1 and 2; *n* = 609)^13^ and an open-label repeat treatment safety study (SAKURA 3; *n* = 2,691, comprising new enrolled subjects and 477 subjects who had rolled over from SAKURA 1 and SAKURA 2)^14^ enrolled adults with moderate or severe glabellar lines at maximum frown. Subjects received 40U DAXI in a standardized 5-point injection pattern into the corrugator and procerus muscles. Glabellar line severity at maximum frown and at rest (after maximum frown) was assessed by investigators using the validated photonumeric Investigator Global Assessment-Frown Wrinkle Severity (IGA-FWS) scale and by subjects using the validated Patient Frown Wrinkle Severity (PFWS) scale. Both the IGA-FWS and PFWS scales grade glabellar line severity from none (0) to severe (3).

In the SAKURA clinical program, there was no enrolment criterion specifically related to static glabellar lines. However, data on the severity of static glabellar lines were collected at baseline and throughout the trials. The current post hoc analysis evaluated the efficacy of repeated DAXI treatment on static glabellar lines among subjects who received 3 DAXI treatment cycles over an 84-week period in the SAKURA clinical program. Subjects were those who were newly enrolled in the SAKURA 3 trial or who were rolled over from the SAKURA 1 and SAKURA 2 trials if their glabellar line severity at maximum frown had returned to baseline on both the IGA-FWS and PFWS scales after 24 weeks. Efficacy was evaluated for up to 36 weeks after DAXI Treatment Cycles 1 and 2 and for up to 12 weeks after DAXI Treatment Cycle 3.

### Outcomes

For the current analysis, efficacy was evaluated by the proportion of subjects with no static glabellar lines at various time points after each DAXI treatment cycle based on either the PFWS or IGA-FWS scale among all subjects who received 3 DAXI treatments. This analysis was repeated among subjects who had at least mild static glabellar lines at baseline (i.e., excluding subjects who had no static glabellar lines at baseline based on either subject or investigator assessment). The proportion of subjects with no static glabellar lines (based on either the PFWS or IGA-FWS scale) according to baseline static glabellar line severity was also determined. In addition, the mean change from baseline in static glabellar line severity at various time points after each DAXI treatment cycle based on the PFWS or IGA-FWS scale among subjects who had at least mild static glabellar lines at baseline (i.e., excluding subjects who had no static glabellar lines at baseline based on either subject or investigator assessment) was evaluated.

### Statistical Analysis

Descriptive statistics were provided for all efficacy outcomes at each time point. For calculation of the proportion of responders, all treated subjects were included in the denominator even if the subjects did not provide data at a given visit.

## Results

### Subject Disposition

Overall, 568 subjects received 3 DAXI treatments in the SAKURA clinical program and were included in the current analysis. This included 228 subjects who rolled over from the SAKURA 1 or SAKURA 2 trials (and received DAXI in these randomized studies) and 340 subjects who were newly enrolled in the SAKURA 3 trial.

### Demographic and Baseline Characteristics

Of the 568 subjects in this analysis, most were White (92.3%) and women (85.7%) and most had at least mild static glabellar lines based on the PFWS scale (91.0%) or the IGA-FWS scale (72.7%) (See **Supplemental Digital Content 1**, Table S1, http://links.lww.com/DSS/A894). The mean age of subjects at baseline tended to increase with increasing static glabellar line severity from 43.3 years for subjects with no static glabellar lines to 55.2 years for subjects with severe static glabellar lines, based on subject assessment, and from 45.4 years for subjects with no static glabellar lines to 54.4 years for subjects with severe static glabellar lines, based on investigator assessment (See **Supplemental Digital Content 2**, Table S2, http://links.lww.com/DSS/A895).

### Efficacy

#### Percentage of Subjects With No Static Glabellar Lines

**Figure 1.**
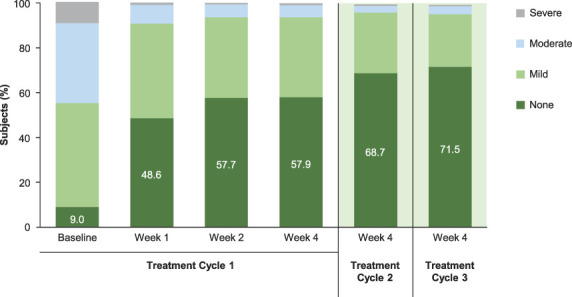
Proportion of subjects with no, mild, moderate, or severe static glabellar lines based on the Patient Frown Wrinkle Severity scale after DaxibotulinumtoxinA for Injection Treatment Cycles 1, 2, and 3.

**Figure 2.**
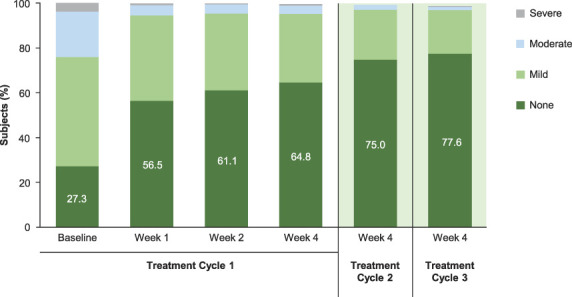
Proportion of subjects with no, mild, moderate, or severe static glabellar lines based on the Investigator Global Assessment-Frown Wrinkle Severity scale after DaxibotulinumtoxinA for Injection Treatment Cycles 1, 2, and 3.

**Figure 3.**
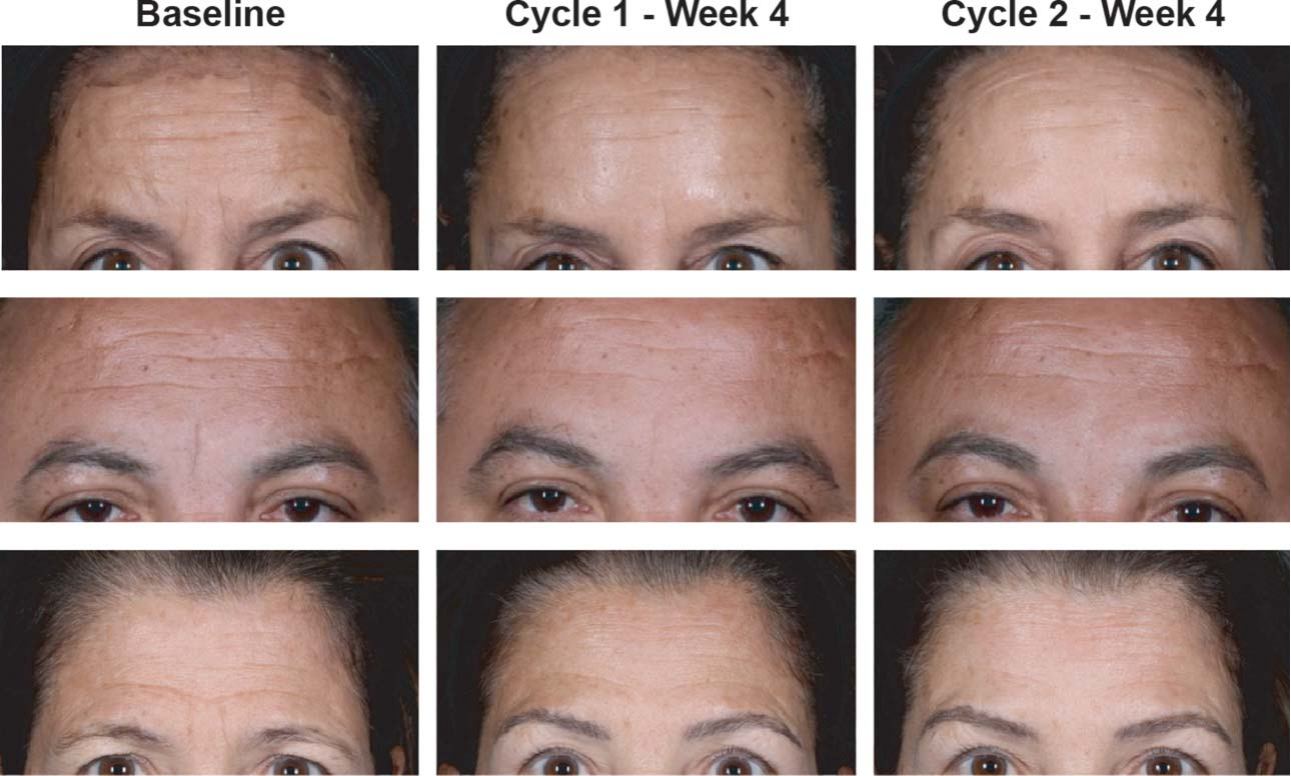
Representative images of static glabellar lines at baseline, Week 4 of DaxibotulinumtoxinA for Injection (DAXI) Treatment Cycle 1, and Week 4 of DAXI Treatment Cycle 2 for 3 individual subjects.

At 4 weeks after DAXI Treatment Cycle 1, the proportion of subjects exhibiting no static glabellar lines increased from 9.0% at baseline to 57.9%, based on subject assessment. At 4 weeks after DAXI Treatment Cycles 2 and 3, the proportion of subjects with no static glabellar lines increased progressively to 68.7% and 71.5%, respectively (Figure 1). A similar progressive improvement was observed based on investigator assessment. At 4 weeks after DAXI Treatment Cycle 1, the proportion of subjects with no static glabellar lines increased from 27.3% at baseline to 64.8%, based on investigator assessment. At 4 weeks after DAXI Treatment Cycles 2 and 3, the proportion of subjects assessed as having no static glabellar lines increased progressively to 75.0% and 77.6%, respectively (Figure 2). Representative images for 3 subjects at rest are shown at baseline and at 4 weeks after DAXI treatment Cycles 1 and 2 in Figure 3.

Similar trends were also observed when subjects with no static glabellar lines at baseline based on subject assessment (i.e., with no ability to demonstrate improvement [*n* = 517]) were excluded from the analyses. At 4 weeks after DAXI Treatment Cycle 1, 54.4% of subjects with mild, moderate, or severe static glabellar lines at baseline had no static glabellar lines based on subject assessment. At 4 weeks after DAXI Treatment Cycles 2 and 3, the proportion of subjects with no static glabellar lines increased to 66.5% and 69.2%, respectively. At the final time point (12 weeks after DAXI Treatment Cycle 3), 51.6% of subjects had no static glabellar lines. Among subjects who had mild, moderate, or severe static glabellar lines at baseline based on investigator assessment (*n* = 413), 53.3% had no static glabellar lines at 4 weeks after DAXI Treatment Cycle 1 and 66.3% and 69.7% of subjects were rated as having no static glabellar lines at 4 weeks after DAXI Treatment Cycles 2 and 3, respectively.

#### Percentage of Subjects Achieving No Static Glabellar Lines According to Baseline Static Glabellar Line Severity

**Figure 4.**
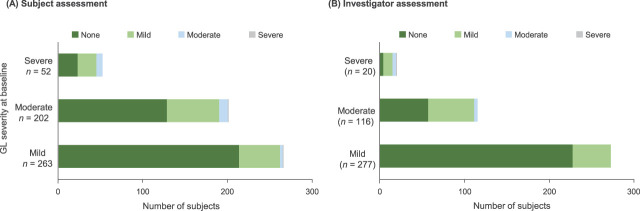
Number of subjects with no static glabellar lines (GLs) at 4 weeks after DaxibotulinumtoxinA for Injection Treatment Cycle 3 according to baseline static GL severity (mild, moderate, or severe) based on (A) subject assessment and (B) investigator assessment.

Based on subject assessment, among subjects with severe static glabellar lines at baseline (*n* = 52), 42% had no static glabellar lines 4 weeks after DAXI Treatment Cycle 3; among those with moderate static glabellar lines at baseline (*n* = 202), 62% had no static glabellar lines at 4 weeks; and among those with mild static glabellar lines at baseline (*n* = 263), 80% had no static lines at 4 weeks (Figure 4A). Similar effects were observed based on investigator assessment (Figure 4B); among those with severe static glabellar lines at baseline (*n* = 20), 20% had no static glabellar lines at 4 weeks after DAXI Treatment Cycle 3; among those with moderate static glabellar lines at baseline (*n* = 116), 49% had no static glabellar lines at 4 weeks; and among those with mild static glabellar lines at baseline (*n* = 277), 82% had no static lines at 4 weeks.

#### Mean Change From Baseline in Static Glabellar Line Severity

An improvement in static glabellar line severity relative to baseline was maintained over the full 24 weeks of follow-up after DAXI Treatment Cycles 1 and 2 based on both PFWS (See **Supplemental Digital Content 3**, Figures S1, http://links.lww.com/DSS/A892) and IGA-FWS scales (See **Supplemental Digital Content 4**, Figure S2, http://links.lww.com/DSS/A893). Importantly, an incremental improvement (greater mean change from baseline) was achieved with each subsequent DAXI treatment cycle. Across all 3 DAXI treatment cycles, the greatest mean change (improvement) from baseline in static glabellar line severity was observed between Weeks 2 and 4 based on the PFWS scale and at Week 4 based on the IGA-FWS scale.

## Discussion

In the current analysis, rapid and sustained improvements in static glabellar line severity were observed after repeated DAXI treatment. Improvements in static glabellar lines were achieved within 2 to 4 weeks after DAXI Treatment Cycle 1, remained greater than baseline over 24 weeks of follow-up after DAXI Treatment Cycles 1 and 2, and more than 70% of subjects had no static glabellar lines 4 weeks after their third DAXI treatment cycle. Furthermore, there was a progressive improvement in glabellar line severity with each subsequent treatment cycle, despite subjects being required to return to baseline dynamic glabellar line severity before each retreatment.

The skin is constantly changing and adapting in response to both internal and external stimuli. In the glabella, repetitive muscle traction on the skin is believed to increase fibrocyte activity resulting in a progressive extracellular remodeling of the hypodermal connective tissue, which further results in the generation of static wrinkles and lines.^3^ When such stimulus is removed after BoNTA treatment, adaptive extracellular changes of the dermal and hypodermal connective tissue result in a smoothing effect and an overall improvement in skin quality.^4,16^ Evidence suggests that regular BoNTA treatments can stimulate collagen production and lead to a reorganization of the collagen network within the extracellular matrix, which may produce more youthful-looking skin.^17–19^ The current study showed that subjects with static glabellar lines at baseline experienced sustained improvements in their static glabellar line severity after retreatment with DAXI. Although the mechanism(s) underlying the observed improvement in static glabellar lines after repeated DAXI treatment have yet to be confirmed, several potential mechanisms may be involved. First, DAXI treatment may minimize or alleviate mechanical stress within the glabellar region, allowing the dermis to lay down collagen and microfibrils.^20^ Second, DAXI treatment may eliminate repetitive skin folding (and therefore chronic stress applied to the dermis), causing collagen and elastin to strengthen over time in these areas.^16^ Third, DAXI treatment weakens the glabellar complex muscles, resulting in unopposed elevation and lifting effect from the (untreated) brow elevators.^21,22^ Finally, DAXI treatment may lead to a combination of effects, which include local muscle relaxation (accounting for early improvements in glabellar lines) and tissue remodeling in response to reduced muscle activity (accounting for later improvements). Although additional studies are needed to better understand the effects of long-term BoNTA treatment on static glabellar line improvement, multiple clinical trials have demonstrated an extended duration of therapeutic benefit with DAXI on dynamic glabellar lines compared with BoNTA treatments currently approved in the United States (6 months vs up to 3–4 months [12–16 weeks] based on product labels).^7,8,11,23^ Given that patients typically only receive 2 BoNTA treatments per year in clinical practice,^9^ patients treated with DAXI are likely to experience a longer duration of effect on dynamic glabellar lines over a year compared with other BoNTAs. Hence, it is possible that this longer duration of therapeutic benefit may provide a longer window of glabellar muscle inactivity (or hypoactivity) and an extended period during which dermal remodeling can occur.

In this study, subjects retained a proportion of their improvement in static glabellar lines over repeated DAXI treatment cycles, which suggests that a long duration of benefit on static glabellar lines at each treatment cycle may lead to a cumulative benefit over time. Although progressive improvements in glabellar line severity relative to baseline with repeated treatment has been reported for onabotulinumtoxinA,^5,24^ subjects in these studies may have been retreated before they returned to their baseline dynamic glabellar line severity. By contrast, subjects in the SAKURA clinical program were required to return to baseline dynamic glabellar lines before retreatment with DAXI. This approach in the SAKURA clinical program thus limited the possibility of a residual treatment effect that may have influenced the observed improvement in static glabellar lines. An analysis of the onabotulinumtoxinA pivotal trials found that subjects with at least mild static glabellar lines at baseline who received 3 treatments with onabotulinumtoxinA 20U at a fixed retreatment interval of 4 months showed an improvement in the severity of static glabellar lines.^5^ In the onabotulinumtoxinA analysis, approximately 55% of subjects had no static glabellar lines at 30 days after Treatment Cycle 1 (based on investigator assessment), whereas in the DAXI analysis, approximately 65% of patients had no static glabellar lines at Week 4 after DAXI Treatment Cycle 1. Although the percentage of responders increased incrementally after onabotulinumtoxinA Treatment Cycles 2 and 3 (with approximately 65% achieving no static glabellar lines after Treatment Cycle 3), the percentage of subjects achieving no static glabellar lines after DAXI Treatment Cycle 3 was markedly greater (approximately 78%). The onabotulinumtoxinA analysis did not evaluate the time course of improvement in static glabellar lines after each treatment; rather, response was defined as achievement of no static glabellar lines at any visit during the treatment cycle. In addition, the timing for repeat onabotulinumtoxinA treatment was predefined (rather than based on individual patient response as in the SAKURA clinical program).

In addition to the added satisfaction patients experience when both dynamic and static glabellar lines are improved at the same time,^25^ these observations of progressive improvement in static glabellar lines over repeated treatment are consistent with previous reports that the effects of BoNTA on static glabellar lines may last longer than the effects on dynamic glabellar lines.^23^ Hence, improvement in static glabellar lines may also contribute to high patient satisfaction after BoNTA treatment, even after dynamic glabellar lines have returned to baseline severity.^26^

There are several limitations for the current analysis. First, the study population in the SAKURA clinical program was primarily White and female. As such, future analyses should aim to include a more diverse study population (e.g., men and patients with a greater diversity of Fitzpatrick skin types). Second, effects on static glabellar lines were evaluated after each of 3 DAXI treatment cycles; following patients over a greater number of treatments may reveal further benefits of long-term continued DAXI treatment on static glabellar lines. Finally, because the units of BoNTA are not interchangeable due to differences in formulation, potency, and the lack of an international reference standard, the units of DAXI in this study are specific to DAXI only and should not be compared with units of other approved BoNTA products. Furthermore, these findings have yet to be confirmed for DAXI in other treatment areas or using other glabellar line dosing schemes.

Overall, the data from the current analysis demonstrate a sustained and progressive improvement in the severity of static glabellar lines over subsequent DAXI treatments. The duration of efficacy of DAXI in glabellar lines likely provides a substantial period of respite from dynamic wrinkle formation in the glabella and consequently permits a greater time for changes to the dermis deep to static, etched-in wrinkles. This is perhaps yet to be appreciated as an advantage of this novel BoNTA product.

## Supplementary Material

SUPPLEMENTARY MATERIAL

## References

[1] Luebberding S Krueger N Kerscher M. Quantification of age-related facial wrinkles in men and women using a three-dimensional fringe projection method and validated assessment scales. Dermatol Surg 2014;40:22–32. 24267416 10.1111/dsu.12377

[2] Fujimura T. Investigation of the relationship between wrinkle formation and deformation of the skin using three-dimensional motion analysis. Skin Res Technol 2013;19:e318–24. 22724632 10.1111/j.1600-0846.2012.00646.x

[3] Piérard GE Lapière CM. The microanatomical basis of facial frown lines. Arch Dermatol 1989;125:1090–2. 2757405 10.1001/archderm.1989.01670200066010

[4] Carruthers A Carruthers J Lei X Pogoda JM . OnabotulinumtoxinA treatment of mild glabellar lines in repose. Dermatol Surg 2010;36(suppl 4):2168–71. 21134048 10.1111/j.1524-4725.2010.01708.x

[5] Carruthers A Carruthers J Fagien S Lei X . Repeated onabotulinumtoxinA treatment of glabellar lines at rest over three treatment cycles. Dermatol Surg 2016;42:1094–101. 27427996 10.1097/DSS.0000000000000704 PMC5414762

[6] Song S Lee YH Hong JP Oh TS. Safety, efficacy, and onset of a novel botulinum toxin type A (Nabota) for the treatment of glabellar frown lines: a single-arm, prospective, phase 4 clinical study. Arch Craniofac Surg 2018;19:168–74. 30282425 10.7181/acfs.2018.01886 PMC6177673

[7] Allergan. BOTOX Cosmetic (OnabotulinumtoxinA) for Injection, for Intramuscular Use Prescribing Information. 2019. Available from: https://media.allergan.com/actavis/actavis/media/allergan-pdf-documents/product-prescribing/20190626-BOTOX-Cosmetic-Insert-72715US10-Med-Guide-v2-0MG1145.pdf.

[8] Merz. XEOMIN (IncobotulinumtoxinA) For Injection, for Intramuscular or Intraglandular Use Prescribing Information. 2019. Available from: https://www.xeominaesthetic.com/wp-content/uploads/2019/05/XEOMIN-Full-Prescribing-Information-including-MedGuide.pdf.

[9] Carruthers A Sadick N Brandt F Trindade de Almeida AR . Evolution of facial aesthetic treatment over five or more years: a retrospective cross-sectional analysis of continuous onabotulinumtoxinA treatment. Dermatol Surg 2015;41:693–701. 25973559 10.1097/DSS.0000000000000340

[10] Evolus. JEUVEAU (PrabotulinumtoxinA-Xvfs) for Injection, for Intramuscular Use Prescribing Information. 2020. Available from: https://info.evolus.com/hubfs/Prescribing%20Info_20200130.pdf

[11] Galderma. DYSPORT (AbobotulinumtoxinA) for Injection, for Intramuscular Use Prescribing Information. 2016. Available from: https://www.accessdata.fda.gov/drugsatfda_docs/label/2016/125274s107lbl.pdf.

[12] Bertucci V Solish N Kaufman-Janette J Yoelin S . DaxibotulinumtoxinA for Injection has a prolonged duration of response in the treatment of glabellar lines: pooled data from two multicenter, randomized, double-blind, placebo-controlled, phase 3 studies (SAKURA 1 and SAKURA 2). J Am Acad Dermatol 2020;82:838–45. 31791824 10.1016/j.jaad.2019.06.1313

[13] Carruthers JD Fagien S Joseph JH Humphrey SD . DaxibotulinumtoxinA for injection for the treatment of glabellar lines: results from each of two multicenter, randomized, double-blind, placebo-controlled, phase 3 studies (SAKURA 1 and SAKURA 2). Plast Reconstr Surg 2020;145:45–58. 31609882 10.1097/PRS.0000000000006327 PMC6940025

[14] Fabi SG Cohen JL Green LJ Dhawan S . DaxibotulinumtoxinA for Injection for the treatment of glabellar lines: efficacy results from SAKURA 3, a large, open-label, phase 3 safety study. Dermatol Surg 2021;47:48–54. 32773446 10.1097/DSS.0000000000002531 PMC7752211

[15] Green JB Mariwalla K Coleman K Ablon G . A large, open-label, phase 3 safety study of DaxibotulinumtoxinA for injection in glabellar lines: a focus on safety from the SAKURA 3 study. Dermatol Surg 2021;47:42–6. 32773447 10.1097/DSS.0000000000002463 PMC7752221

[16] Bonaparte JP Ellis D. Skin biomechanical changes after injection of onabotulinum toxin A: prospective assessment of elasticity and pliability. Otolaryngol Head Neck Surg 2014;150:949–55. 24664544 10.1177/0194599814526558

[17] Humphrey S Jacky B Gallagher CJ. Preventive, cumulative effects of botulinum toxin type A in facial aesthetics. Dermatol Surg 2017;43:S244–51. 33065950 10.1097/DSS.0000000000001404

[18] Chang SP Tsai HH Chen WY Lee WR . The wrinkles soothing effect on the middle and lower face by intradermal injection of botulinum toxin type A. Int J Dermatol 2008;47:1287–94. 19126019 10.1111/j.1365-4632.2008.03895.x

[19] El-Domyati M Attia SK El-Sawy AE Moftah NH . The use of botulinum toxin-a injection for facial wrinkles: a histological and immunohistochemical evaluation. J Cosmet Dermatol 2015;14:140–4. 25916463 10.1111/jocd.12144

[20] Bowler PJ. Dermal and epidermal remodeling using botulinum toxin type A for facial, non reducible, hyperkinetic lines: two case studies. J Cosmet Dermatol 2008;7:241–4. 18789065 10.1111/j.1473-2165.2008.00399.x

[21] Carruthers A Carruthers J. Eyebrow height after botulinum toxin type A to the glabella. Dermatol Surg 2007;33:S26–31. 17241411 10.1111/j.1524-4725.2006.32328.x

[22] Petchngaovilai C. Midface lifting with botulinum toxin: intradermal technique. J Cosmet Dermatol 2009;8:312–6. 19958437 10.1111/j.1473-2165.2009.00467.x

[23] Glogau R Kane M Beddingfield F Somogyi C . OnabotulinumtoxinA: a meta-analysis of duration of effect in the treatment of glabellar lines. Dermatol Surg 2012;38:1794–803. 23106853 10.1111/j.1524-4725.2012.02582.x

[24] Dailey RA Philip A Tardie G. Long-term treatment of glabellar rhytides using onabotulinumtoxina. Dermatol Surg 2011;37:918–28. 21575099 10.1111/j.1524-4725.2011.02024.x

[25] Patel MP Talmor M Nolan WB. Botox and collagen for glabellar furrows: advantages of combination therapy. Ann Plast Surg 2004;52:442–7. 15096921 10.1097/01.sap.0000123806.03865.4d

[26] Rivers JK Bertucci V McGillivray W Muhn C . Subject satisfaction with onabotulinumtoxinA treatment of glabellar and lateral canthal lines using a new patient-reported outcome measure. Dermatol Surg 2015;41:950–9. 26218728 10.1097/DSS.0000000000000424

