# Supplementary material for: Progressive Improvement in Static Glabellar Lines After Repeated Treatment With DaxibotulinumtoxinA for Injection
Source: Dermatol Surg. 2021 Aug 16;47(12):1579–84. doi: 10.1097/DSS.0000000000003211 (PMC8612903; doi:10.1097/DSS.0000000000003211)
Supplement: SUPPLEMENTARY MATERIAL [file ds-47-1579-s001.docx]

**Table S1. Demographic and Baseline Characteristics**

|  | **Subjects Who Received**  **3 DAXI Treatments, (*N* = 568)** |
| --- | --- |
| Age, mean (SD), years | 50.7 (10.2) |
| Sex, female, *n* (%) | 487 (85.7) |
| Race, *n* (%) | |
| White | 524 (92.3) |
| Black/African American | 16 (2.8) |
| Asian | 16 (2.8) |
| Other^a^ | 12 (2.1) |
| Static glabellar line severity based on PFWS scale, *n* (%) | |
| None | 51 (9.0) |
| Mild | 263 (46.3) |
| Moderate | 202 (35.6) |
| Severe | 52 (9.2) |
| Static glabellar line severity based on IGA-FWS scale, *n* (%) | |
| None | 155 (27.3) |
| Mild | 277 (48.8) |
| Moderate | 116 (20.4) |
| Severe | 20 (3.5) |

^a^Includes Native Hawaiian/Other Pacific Islander, American Indian or Alaska Native, Multiple and Other.

IGA-FWS, Investigator Global Assessment-Frown Wrinkle Severity; PFWS, Patient Frown Wrinkle Severity.
