# Supplementary material for: Progressive Improvement in Static Glabellar Lines After Repeated Treatment With DaxibotulinumtoxinA for Injection
Source: Dermatol Surg. 2021 Aug 16;47(12):1579–84. doi: 10.1097/DSS.0000000000003211 (PMC8612903; doi:10.1097/DSS.0000000000003211)
Supplement: SUPPLEMENTARY MATERIAL [file ds-47-1579-s002.docx]

**Figure S1.** Mean change from baseline in static glabellar line severity following DAXI Treatment Cycles 1, 2, and 3 based on Patient Frown Wrinkle Severity Scale (0 = none to 3 = severe).

**
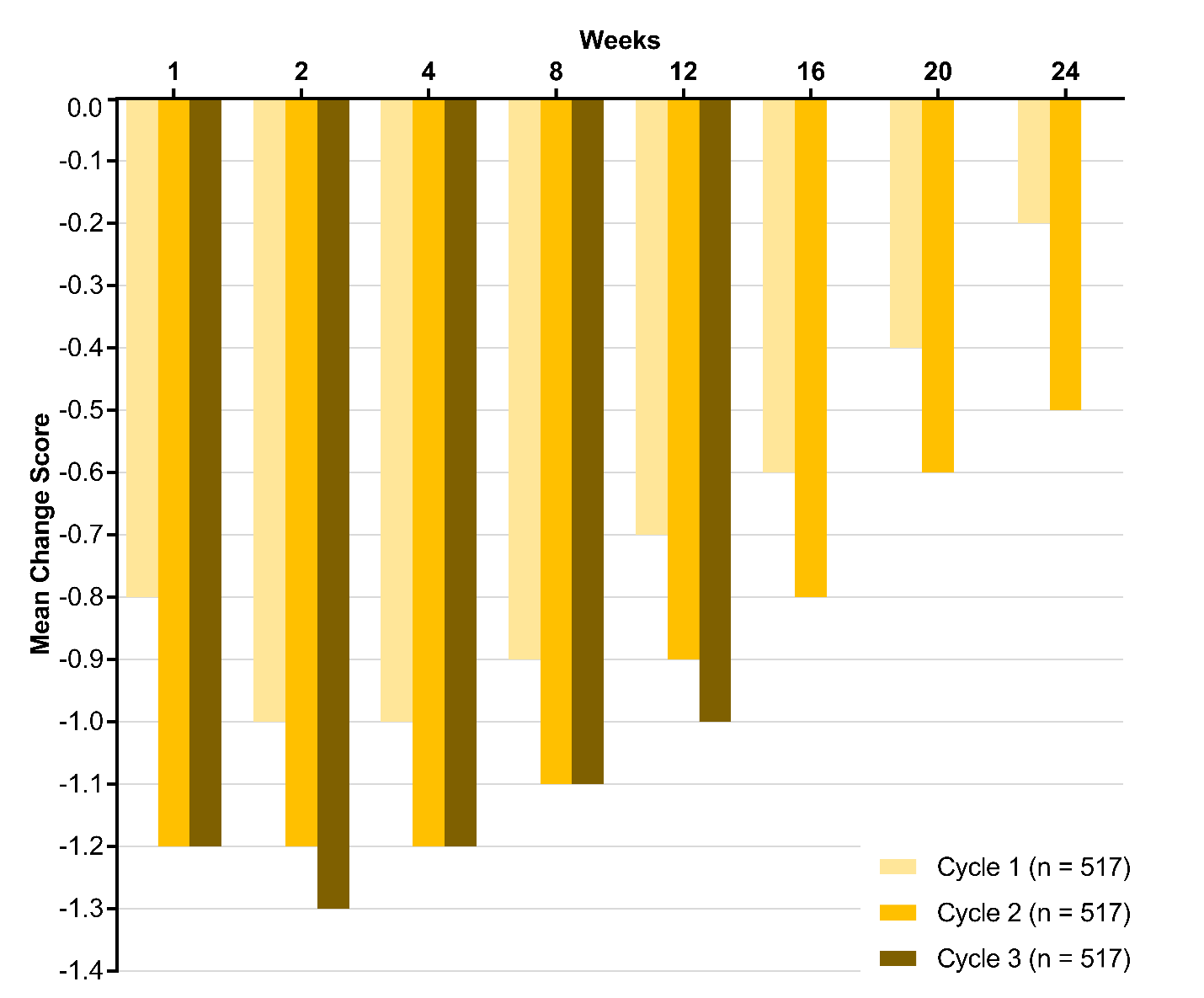
**
