# Supplementary material for: Progressive Improvement in Static Glabellar Lines After Repeated Treatment With DaxibotulinumtoxinA for Injection
Source: Dermatol Surg. 2021 Aug 16;47(12):1579–84. doi: 10.1097/DSS.0000000000003211 (PMC8612903; doi:10.1097/DSS.0000000000003211)
Supplement: SUPPLEMENTARY MATERIAL [file ds-47-1579-s003.docx]

**Table S2. Demographic and Baseline Characteristics Based on Baseline Static Glabellar Line Severity as Assessed by the Subject Via the Patient Frown Wrinkle Severity Scale and the Investigator Via the Investigator Global Assessment-Frown Wrinkle Severity Scale**

|  | **PFWS** | | | | **IGA-FWS** | | | |
| --- | --- | --- | --- | --- | --- | --- | --- | --- |
|  | **None (*n* = 51)** | **Mild (*n* = 263)** | **Moderate (*n* = 202)** | **Severe (*n* = 52)** | **None (*n* = 155)** | **Mild (*n* = 277)** | **Moderate (*n* = 116)** | **Severe (*n* = 20)** |
| Age, mean (SD), years | 43.3 (10.8) | 50.3 (10.0) | 52.1 (9.6) | 55.2 (8.7) | 45.4 (11.3) | 52.5 (9.2) | 52.9 (8.6) | 54.4 (5.6) |
| Sex, female, n (%) | 40 (78.4) | 228 (86.7) | 175 (86.6) | 44 (84.6) | 133 (85.8) | 243 (87.7) | 101 (87.1) | 10 (50.0) |
| Race, n (%) | | | | | | | | |
| White | 44 (86.3) | 243 (92.4) | 186 (92.1) | 51 (98.1) | 140 (90.3) | 254 (91.7) | 111 (95.7) | 19 (95.0) |
| Black/African American | 2 (3.9) | 5 (1.9) | 9 (4.5) | 0 | 6 (3.9) | 7 (2.5) | 2 (1.7) | 1 (5.0) |
| Asian | 3 (5.9) | 8 (3.0) | 4 (2.0) | 1 (1.9) | 5 (3.2) | 11 (4.0) | 0 | 0 |
| Other^a^ | 2 (3.9) | 7 (2.7) | 3 (1.5) | 0 | 4 (2.6) | 5 (1.8) | 3 (2.6) | 0 |

^a^Includes Native Hawaiian/Other Pacific Islander, American Indian or Alaska Native, Multiple and Other.

IGA-FWS, Investigator Global Assessment-Frown Wrinkle Severity; PFWS, Patient Frown Wrinkle Severity.
